# Pleural epithelioid angiosarcoma with lymphatic differentiation arisen after radiometabolic therapy for thyroid carcinoma: immunohistochemical findings and review of the literature

**DOI:** 10.1186/s13000-017-0652-1

**Published:** 2017-08-15

**Authors:** Daniela Cabibi, Giulia Pipitone, Rossana Porcasi, Sabrina Ingrao, Ignazio Benza, Calogero Porrello, Massimo Cajozzo, Antonino Giulio Giannone

**Affiliations:** 1 Department of Sciences for the Promotion of Health and Mother and Child Care, Anatomic Pathology, University of Palermo, Palermo, Italy; 2 Buccheri La Ferla Hospital, Unit of Radiology, Palermo, Italy; 3 Department of Surgical and Oncological Sciences, University of Palermo, Palermo, Italy; 4 Anatomia Patologica, A.O.U. Policlinico ‘P. Giaccone’, Via del Vespro 129, 90127 Palermo, Italy

**Keywords:** Pleural angiosarcoma, Radiometabolic therapy, Immunohistochemistry, Thyroid Carcinoma

## Abstract

**Background:**

Pleural angiosarcoma is a rare tumor that causes diffuse pleural thickening and effusion, mimicking mesothelioma. Immunohistochemistry is needed to highlight endothelial differentiation. We describe the first case of pleural angiosarcoma with lymphatic differentiation following radiometabolic therapy for thyroid carcinoma.

**Case presentation:**

A 50-year-old man showed diffuse pleural thickening and effusion. Nine years earlier, he underwent thyroidectomy and radiometabolic therapy for thyroid carcinoma with lymph node metastases. Histologically, the tumor consisted of a solid proliferation of atypical epithelioid cells and anastomosed vascular spaces, lacking of red blood cells and containing Alcian blue positive material. The tumor showed positive immunostaining for Vimentin, CD31, CK7, D2–40, c-MYC, Ki67, focal positivity for PanCK, and negative immunostaining for Factor VIII, CD34, WT1, CK5/6, Calretinin, EMA, HBME-1, CEA, p63, EpCAM, Bcl-2, TTF1 and Thyroglobulin. CD99 showed a granular/paranuclear pattern of positivity. The histological and immunohistochemical features were consistent with “pleural angiosarcoma with lymphatic differentiation, epithelioid variant”.

**Discussion and conclusions:**

Epithelioid angiosarcoma with lymphatic differentiation is very rare and aggressive. Moreover, the positivity for c-MYC suggests the relationship with radiometabolic therapy. To our knowledge, this is the first case of pleural c-MYC-positive angiosarcoma with lymphatic differentiation reported in the literature and the first one arisen after radiometabolic therapy for thyroid carcinoma.

## Background

Angiosarcoma (AS) is a malignant neoplasm with endothelial differentiation, accounting for approximately 1–2% of all soft tissue sarcomas. Pleura represents an exceedingly rare site for AS, with about 50 cases reported in the literature [1]. Patients almost always die from the disease within months. The diagnosis of this rare pleural tumor is challenging, mimicking mesothelioma, pulmonary carcinoma, or metastatic adenocarcinoma, due to non-specific clinical and radiologic findings and to the positivity for epithelial markers, such as pan-cytokeratins (PanCK) and CK7. Markers of endothelial differentiation, such as CD31, CD34 and Factor VIII, are particularly useful in the identification of these tumors [2, 3].

Lymphangiosarcoma (LAS) is even rare. Both AS and LAS typically arise about 10 years after axillary nodal dissection or radiation therapy for breast cancer with long-standing massive lymphedema [4] and have been reported in literature as Stewart-Treves Syndrome. LAS has been also evidenced after chronic lymphedema of lower leg [5]. In serous membranes, few cases of LAS have been reported after therapeutic irradiation [6].

We describe a case of primary pleural epithelioid AS with lymphatic differentiation, clinically and radiologically suspected as mesothelioma, arisen 9 years after radiometabolic therapy (RMT) for thyroid papillary carcinoma with multiple lymph node metastases. To the best of our knowledge, only one case of pleural epithelioid LAS has been previously reported [1]. It is noteworthy that this is the first reported case of pleural epithelioid AS with lymphangioendothelial differentiation arisen after RMT for thyroid carcinoma.

## Case report

A 50 year-old male patient with a history of dyspnea was admitted to our hospital.

### Past medical history

Nine years earlier, in another hospital, the patient underwent total thyroidectomy for papillary carcinoma with dissection of the back-left jugular lymph nodes, one of which metastatic.

The tumor was located in the upper half of the left lobe and had a maximum diameter of 4,5 cm. The histologic examination revealed a well differentiated papillary carcinoma, classical variant, with multiple microscopic foci in both lobes of the thyroid gland; the tumor stained positively for TTF1 and Thyroglobulin. One out of 8 lymph nodes was metastatic.

After six months, PET and total body scintigraphy evidenced the presence of lymph node metastases on the left side of the neck (level III and IV), and the patient underwent RMT with iodine-131 (^131^I, 100 mCi).

Subsequently, he was subjected to surgical lymphadenectomy of 22 cervical and supraclavicular lymph nodes, three of which metastatic. Due to the persistence of residual lymph node metastases, he underwent three cycles of RMT (100 mCi; 3,7GBq orally). So, he received a cumulative dose of 400 mCi. It is noteworthy that before the third cycle of RMT, the total body scintigraphy evidenced low and widespread ^131^I uptake in both lungs and in the para-medial left mediastinal area, leading to the suspicion of mediastinal and lung involvement. Thoracic PET and CT did not evidence nodular lesions, ruling out the suspicion of metastasis.

Finally, a new lymphadenectomy was performed, due to the persistence of metastases in 2/4 retro-clavear lymph nodes, in 4/22 level II left lymph nodes and in level III left lymph nodes.

### Current medical history

**Fig. 1:**
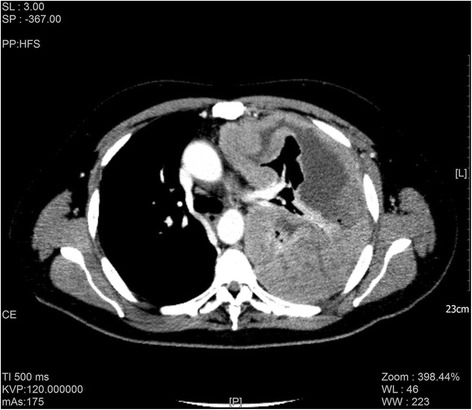
CT image revealed irregular circumferential pleural thickening with nodular soft tissue masses, involving the mediastinal and diaphragmatic pleura, and extending into the fissures, with reduction of right lung volume

The chest x-ray showed an opaque left hemithorax, saving a small central lung area with contralateral cardio-mediastinal shift [Fig. 1]. CT images showed irregular circumferential pleural thickening with nodular soft tissue masses involving the mediastinal and diaphragmatic pleura, extending into the fissures and reducing the volume of the right lung. These features clinically suggested pleural mesothelioma, and multiple biopsies of the left parietal pleura were performed during Video-Assisted Thoracotomy (VATS).

**Fig. 2:**
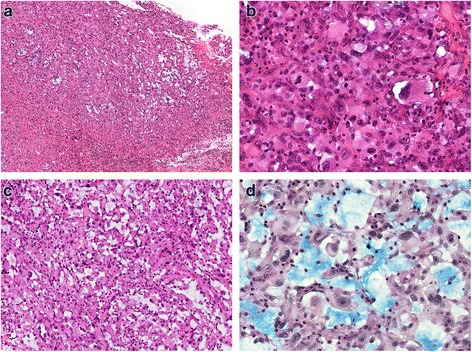
**a** On low power magnification, the tumor consisted of “vasoformative” areas admixed with more solid areas. **b** Solid areas constituted by highly atypical epithelioid cells. **c** “Vasoformative” areas characterized by vascular channels lined by plumped atypical cells, lacking of red blood cells. **d** Vascular channels containing Alcianblue positive mucoid substance. **a**: 40×, H-E; **b**: 200×, H-E; **c**: 100×, H-E; **d**: Alcian-PAS, 200×

**Fig. 3:**
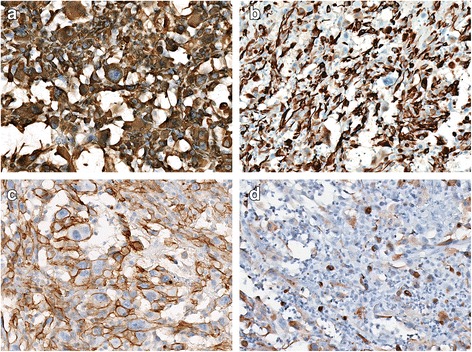
Positive immunostaining for Vimentin (**a**), CK7 (**b**), D2–40 (**c**) Focal positive immunostaining for PanCK (**d**). **a**-**d**: 200×, immunoperoxidase stain

**Fig. 4:**
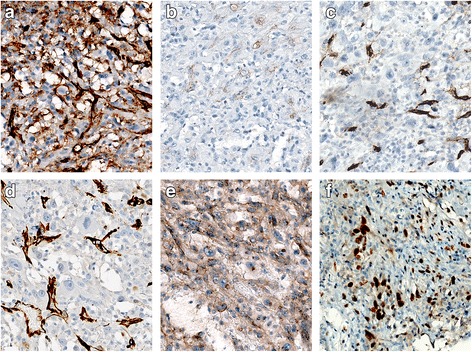
**a** Positive immunostaining for CD31. **b**-**c** Negative immunostaining for CD34 (**b**) FVIII (**c**) and WT1 (**d**). A few non-neoplastic positive vascular spaces have been used as internal positive control. **e** Vacuolar/paranuclear positive pattern of CD99. **f** Positive nuclear immunostaining for c-MYC. **a**-**f**: 200×, immunoperoxidase stain

**Fig. 5:**
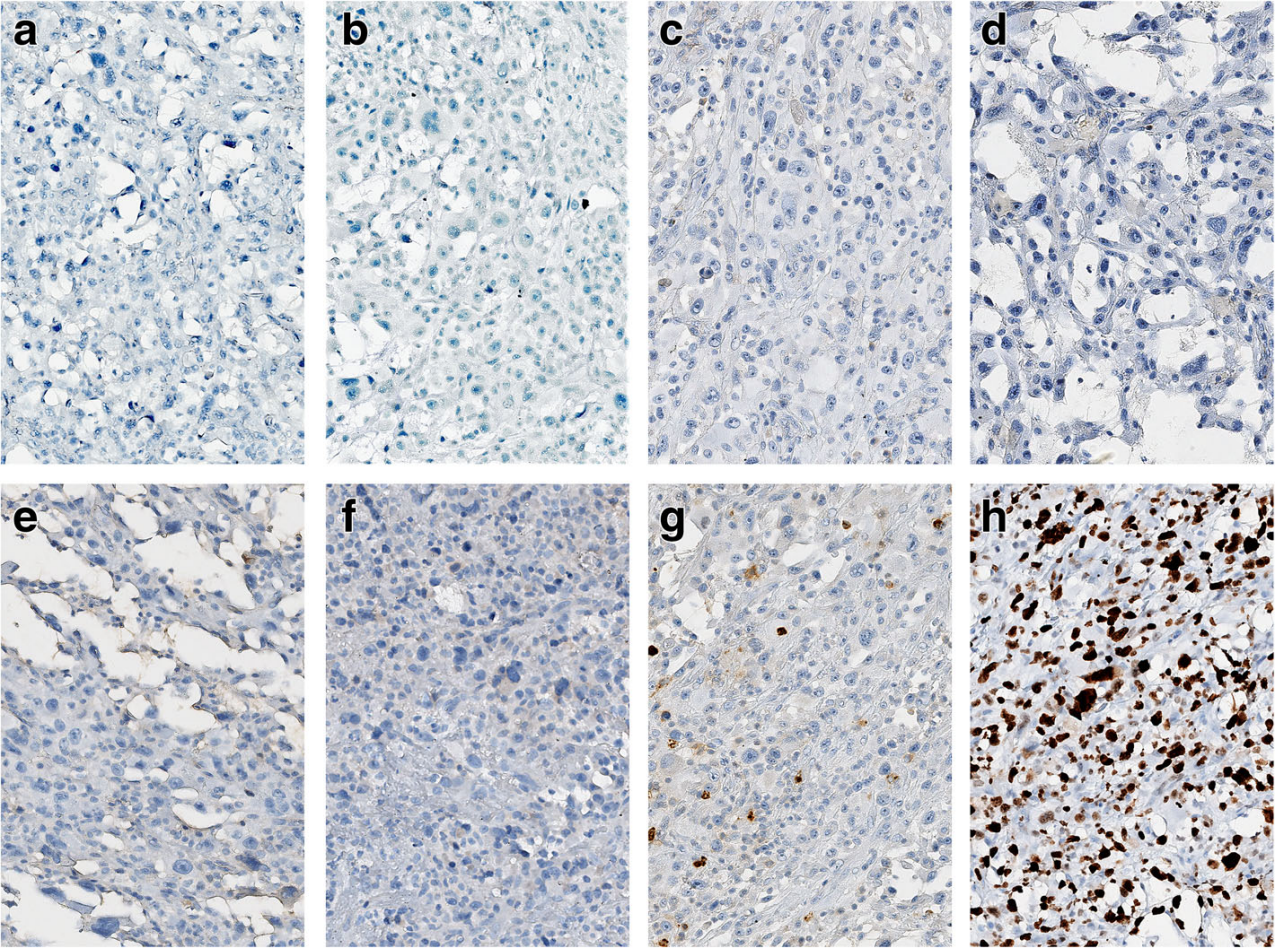
Negative immunostaining for Calretinin (**a**), CK5/6 (**b**), EMA (**c**), HBME-1 (**d**) TTF-1 (**e**), Thyroglobulin (**f**), CEA (**g**). Positive nuclear immunostaining for Ki67 (**h**). **a**-**h**: 200×, immunoperoxidase stain

On microscopic examination, the hematoxylin-eosin (H-E) sections of formalin-fixed paraffin-embedded biopsies showed a neoplastic proliferation of highly atypical epithelioid cells with mitoses and areas of necrosis. The tumor was composed of solid areas [Fig. 2a, b] and “vasoformative” areas with variably sized anastomotic vascular spaces, lined by atypical endothelial cells with plump and prominent nuclei. Most of the vascular spaces were lacking of red blood cells and contained certain amounts of Alcian blue positive material [Fig. 2c, d]. The immunohistochemical analysis showed positive immunostaining for Vimentin, CK7, D2–40 and focally for PanCK [Fig. 3a-d], both in the vascular and in the solid areas. The vascular endothelial lining showed focal positivity for CD31 [Fig. 4a].CD34, Factor VIII related-antigenand WT1 stained positively only in a few non-neoplastic vascular channels that were used as positive internal control [Fig. 4b-d]. CD99 showed a paranuclear pattern and/or granular pattern of positivity [Fig. 4e]. Nuclei of many tumor cells stained positively for c-MYC [Fig. 4f]. Calretinin, CK5/6, EMA, HBME-1, TTF-1, Thyroglobulin and CEA stained negatively [Fig. 5a-g]. The expression of Ki67 was high, with about 30% of positive neoplastic nuclei [Fig. 5h]. Finally, negative immunostaining was evidenced for p63, Bcl-2, EpCAM and LNA-1 HHV8. The overall morphologic and immunohistochemical features supported the diagnosis of pleural malignant epithelioid vascular tumor, consistent with epithelioid AS with lymphatic differentiation.

## Discussion

Pleural AS is a rare malignant neoplasm composed of atypical and pleomorphic endothelial cells lining abnormal vascular spaces. The radiologic features are nonspecific and usually show unilateral or bilateral effusion and diffuse pleural thickening that can easily simulate mesothelioma or metastases. Mass lesions are seen in about half of the patients [2, 3]. There are two histological variants of pulmonary/pleural AS: classical and epithelioid. Classical AS exhibits a “vasoformative” pattern, with irregular anastomotic vascular spaces containing red blood cells, lined by atypical endothelial cells. “Vasoformative” component is focal in epithelioid AS, that instead shows a predominant solid growth pattern composed of large, round to polygonal epithelioid cells with abundant eosinophilic cytoplasm, large pleomorphic nuclei and prominent nucleoli. Epithelioid features are mentioned in the majority of pleural AS (72,5%) and constitute a variable proportion of the tumors [2, 7].

Immunohistochemical staining play an important role in the differential diagnosis. Our case consisted of both solid epithelioid areas and anastomosing vascular areas. It showed co-expression of Vimentin, CK7, D2–40, focal PanCK, but it stained negatively for EMA, CEA, EpCAM, usually positive in sarcomatoid carcinoma. The negative immunostaining for EMA and for markers usually positive in mesothelioma, such asCK5/6, Calretinin, HBME1, together with the positive immunostaining for CD31, were useful in the differential diagnosis with mesothelioma. CD31 in fact is a marker of vascular lineage, more sensible and specific than CD34, WT1, FVIII, and it is almost always negative in non-vascular neoplasms, such as mesotheliomas and carcinomas [3, 8, 9].

Among the vascular neoplasias, the differential diagnosis between angiosarcoma and epithelioid hemangioendothelioma can be very challenging. Epithelioid hemangioendothelioma typically arises from blood vessels with a characteristic appearance at low-power magnification and it is composed of short strands or solid nests of epithelioid endothelial cells. Distinct vascular spaces are rarely observed and more frequently the tumor cells form small intracellular lumens. Conversely, epithelioid angiosarcomas are composed of solid sheets of highly atypical, mitotically active epithelioid cells of endothelial lineage. Necrosis is common, and vascular differentiation is evidenced by the formation of irregular sinusoidal vascular channels in the “vasoformative” areas [10].

Some authors reported that grade 3 AS could co-express both blood and lymphatic markers [11]. On the other side, Kahn et al. stated that D2–40 stains all benign lymphatic tumors but not benign proliferations of blood vessel origin. So, they concluded that “a subset of AS with positive D2-40 cells can bear partial differentiation along the lymphatic endothelial lineage and should be classified as LAS” [12].

In our case, the presence of sinusoidal vascular channels lacking of red blood cells and containing amorphous Alcian blue positive substance, the marked cellular atypia, the presence of necrosis and numerous atypical mitoses, the high Ki67 expression and the positive immunostaining for CD31 and D2–40 were more consistent with the diagnosis of “epithelioid angiosarcoma with lymphatic differentiation”.

Finally, for both the morphological and immunohistochemical features it is worth noting the negative immunostaining for TTF1 and thyroglobulin. This makes unlikely to assume metastasis from the well differentiated papillary carcinoma, that stained positively for TTF1 and thyroglobulin and surgically removed nine years earlier. Although the expression of CD99 has been reported in several malignant neoplasias and it is not specific, the focal expression of CD99 was in keeping with previous studies highlighting CD99 staining in 25% of the epithelioid sarcomas [13].

AS represents a well-known complication in the setting of chronic lymphedema (Stewart-Treves Syndrome) and after radiotherapy for breast carcinoma, carcinoma of the vulva and Hodgkin lymphoma [14–16]. Primary pleural AS is very rare and the pathogenesis is still unknown. Recently, Zhang et al. described a case of “de novo pleural epithelioid AS” and they found only 18 cases of primary AS of the pleura previously reported in the literature [17].

Some case reports from Japan indicate the relationship with chronic tuberculous pyothorax [18, 19]. A history of exposure to radiation or asbestos was noted in a few Western cases [3]. Our patient was repeatedly subjected to lymphadenectomy but he never showed signs of lymphatic stasis. It is noteworthy that during the treatment, a total-body scintigraphy evidenced a low and widespread uptake of ^131^I in both lungs and in the para-medial left mediastinal area.

Moreover, the positivity for c-MYC is in keeping with recent studies reporting that positive nuclear immunostaining for c-MYC “can reliably discriminate between AS secondary to irradiation and AS arising de novo, that are morphologically indistinguishable” [20–23].

Previous studies suggested that the exposure to ionizing radiation from radioisotope therapy or external local radiotherapy raises the risk of developing second primary cancers, and the risk increases with cumulative doses of ^131^I [24–29]. Our patient received a ^131^I cumulative dose of 400 mCi and he died four months after the diagnosis.

## Conclusions

In this case of c-MYC positive pleural angiosarcoma with lymphatic differentiation, a pathogenetic relationship between the pleural AS and the oncogenic effects of the ionizing radiations could be hypothesized. To the best of our knowledge, this is the first case of pleural AS with lymphatic differentiation following radiometabolic exposure for metastatic thyroid papillary carcinoma and the first pleural AS with lymphatic differentiation in which c-MYC positivity has been highlighted.

## Abbreviations

AS: Angiosarcoma
CEA: Carcino-embrionic antigen
CK: Cytokeratin
EMA: Epithelial membrane antigen
HBME-1: Hector Battifora Mesothelial-1
H-E: Hematoxylin and eosin
LAS: Lymphangiosarcoma
RMT: Radiometabolic therapy
TTF1: Thyroid transcription factor
VATS: Video-Assisted Thoracotomy
WT1: Wilms Tumor-1
